# Influence of Altered Thyroid Hormone Mechanisms in the Progression of Metabolic Dysfunction Associated with Fatty Liver Disease (MAFLD): A Systematic Review

**DOI:** 10.3390/metabo12080675

**Published:** 2022-07-22

**Authors:** Rafael Aguiar Marschner, Fernanda Arenhardt, Rafael Teixeira Ribeiro, Simone Magagnin Wajner

**Affiliations:** 1 Thyroid Section, Endocrine Division, Hospital de Clínicas de Porto Alegre, Universidade Federal do Rio Grande do Sul (UFRGS), Porto Alegre 90035-003, RS, Brazil; rafamarschner@gmail.com (R.A.M.); farenhardt@gmail.com (F.A.); 2 Institute of Basic Health Sciences, Universidade Federal do Rio Grande do Sul (UFRGS), Porto Alegre 90035-003, RS, Brazil; rafaelrated@hotmail.com; 3 Department of Internal Medicine, Universidade Federal do Rio Grande do Sul (UFRGS), Porto Alegre 90035-003, RS, Brazil

**Keywords:** thyroid hormones, liver, MAFLD

## Abstract

We performed a systematic review of the mechanisms of thyroid hormones (THs) associated with metabolic dysfunction associated with fatty liver disease (MAFLD). This systematic review was registered under PROSPERO (CRD42022323766). We searched the MEDLINE (via PubMed) and Embase databases from their inception to March 2022. We included studies that assessed thyroid function by measuring the serum level of THs and those involved in MAFLD. We excluded reviews, case reports, editorials, letters, duplicate studies and designed controls. Forty-three studies included MAFLD, eleven analyzed THs, and thirty-two evaluated the mechanisms of THs in MAFLD. Thyroid hormones are essential for healthy growth, development and tissue maintenance. In the liver, THs directly influence the regulation of lipid and carbohydrate metabolism, restoring the homeostatic state of the body. The selected studies showed an association of reduced levels of THs with the development and progression of MAFLD. In parallel, reduced levels of T3 have a negative impact on the activation of co-regulators in the liver, reducing the transcription of genes important in hepatic metabolism. Overall, this is the first review that systematically synthesizes studies focused on the mechanism of THs in the development and progression of MAFLD. The data generated in this systematic review strengthen knowledge of the impact of TH changes on the liver and direct new studies focusing on therapies that use these mechanisms.

## 1. Introduction

Thyroid hormones (THs) are crucial for of all systems through the human body to work properly. Although thyroxine (T_4_) is the main product of the thyroid, the biologically active hormone is triiodothyronine (T_3_). Both THs enter cells through specific transporters and are subsequently regulated by the activity of the type 1, 2 and 3 iodothyronine deiodinases family [1], which activates (D1 and D2) and inactivates (D3) both T3 and T4 [2]. THs play a fundamental role in liver metabolism through a well-known metabolic network [3]. They directly influence lipid and carbohydrate metabolism in hepatocytes, maintaining plasma levels of triglycerides (TG), low-density lipoprotein (LDL) and high-density lipoprotein (HDL) cholesterol [4], leading to hepatic lipogenesis, lipid oxidation and gluconeogenesis [5,6]. The circulating levels of THs are directly associated with liver metabolism [7]. Evidence has shown that lower availability of T_3_ or dysfunction of thyroid hormone receptors (THRs) leads to a reduction in free fatty acid (FFA) uptake by hepatocytes, lower mitochondrial β-oxidation and changes the lipogenesis processes. 

In recent decades, MAFLD was recognized as the most common form of chronic liver disease, particularly in industrialized countries due to increased obesity in this population [8,9]. It is estimated that the prevalence of MAFLD is around 20–30% in the Western countries and 5–18% in the East [10]. MAFLD is a complex and multifactorial disease, with genetic, epigenetic and environmental factors involved in its pathogenesis. The alterations begin with the deposit of fat in the liver (secondary to a high fat diet (HFD)). The excess of fat leads to lipotoxicity, generating chronic inflammation. MAFLD then progress to more advanced stages such as non-alcoholic steatohepatitis (NASH), characterized by high levels of oxidative stress and increased damage to liver tissue. Next, the hepatic tissue deposits collagen in the injured cells in an attempt to reduce the damage generated, leading to the fibrosis phase. If there is no change, the next stage is cirrhosis and sometimes hepatocellular carcinoma (HCC) [9,11,12].

Previous studies showed that serum levels of THs are associated with the start and progression of metabolic dysfunction associated with fatty liver disease (MAFLD) [11,12,13]. The main association was between hypothyroidism and MAFLD, leading to increased risk of hepatic fibrosis in the long term. However, the exact mechanisms that orchestrate thyroid hormone regulation of hepatic metabolism are still unclear when related to the progression to more severe cases of the disease. Thus, this systematic review aimed to better understand the newly recognized mechanisms involved in the dysfunctional metabolism of THs in MAFLD progression.

## 2. Results

### 2.1. Identified Records

**Figure 1:**
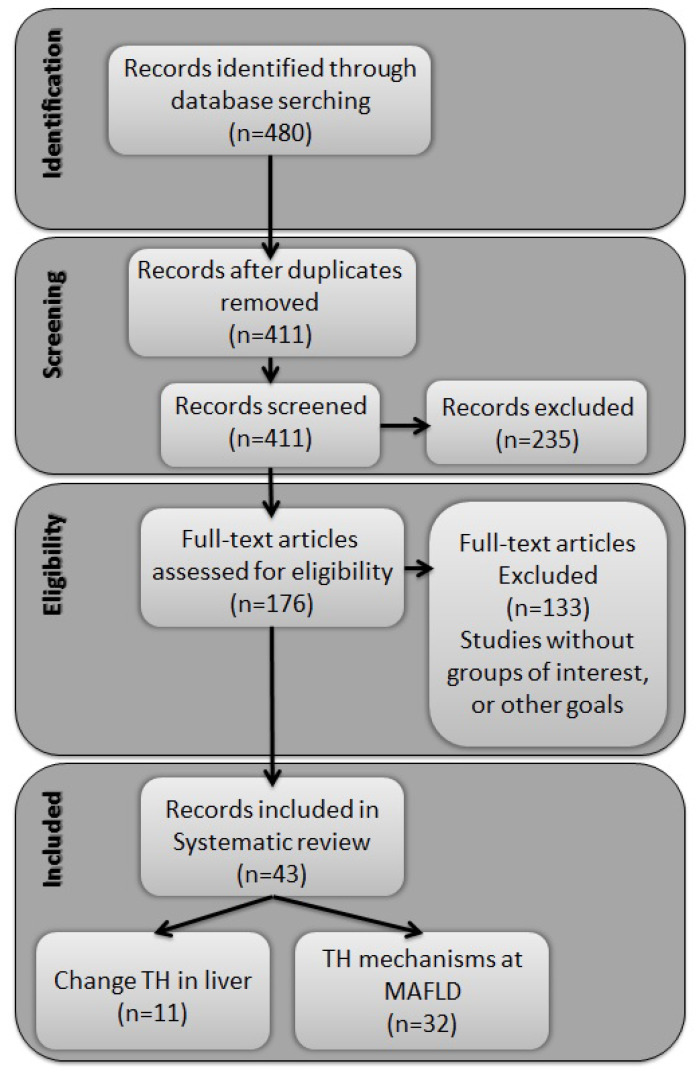
Flowchart of study selection.

Our search initially identified 480 titles and abstracts of potentially eligible studies through database searching. After duplicate removal, 411 records were screened, and 176 full texts were assessed for eligibility. Of these, 133 did not provide information about the outcome of interest. Forty-three studies were included in the analysis, eleven related to changes in serum levels of THs and the liver, and thirty-two evaluating alterations in the metabolism of THs in relation to the progression of MAFLD (Figure 1).

### 2.2. Altered Circulating Levels of Thyroid Hormone and the Liver

The studies selected in Table 1 verified the effect of serum levels of thyroid hormones on the liver, mainly related to the progression of MAFLD. Eleven articles evaluated the serum levels of THs in the context of MAFLD. Of these, one developed an experimental model [14] and the other ten were clinical studies. Among the clinical studies, most had a cross-sectional or retrospective design; only one study was of a prospective cohort [15].

The selected clinical articles used two types of MAFLD assessment technique. Seven were based on imaging tests, while three studies performed biopsies, considered the gold standard for the diagnosis of the disease. The TH groups were separated into euthyroid, hypothyroid or hyperthyroid. With respect to the liver profile, groups were divided into with or without MAFLD, fibrosis or steatosis. One study also verified the action of type 2 diabetes (DM2) on MAFLD and levels THs [16]. All studies demonstrated an association between hypothyroidism with the development of MAFLD.

One study verified the hepatic effects of hyperthyroidism [14]. Interestingly, the authors suggest that increasing the availability of T3 protects, to some extent, the liver from disease progression. However, as it is an experimental study it is not possible to draw definitive conclusions, and further studies are necessary to assess this hypothesis. Another study evaluated gene mutation of the TH receptor, THR-β [17]. A decrease in the availability of the receptor in the liver tissue also increased the risk of developing MAFLD.

The data in Table 1 is a compilation of these results and demonstrates an overall direct association between hypothyroidism and MAFLD. Fat deposition in the liver increases in situations of low serum T3 levels. A long-term, increased risk for MAFLD progression to more severe stages, such as fibrosis, cirrhosis, and hepatocellular carcinoma can be considered when the hormone is not supplemented to euthyroid levels.

**Table 1:** Changes in thyroid hormones and their effects on the liver.

Manuscript	Sample	Study Design	MAFLD Assessment Technique	Groups	Serum Levels of THs	Effect on Liver
Klieverik et al. [14] (2009)	Rats	—	—	G1: Euthyroid G2: Hypothyroidism G3: Thyrotoxic	G2: ↓ THs G3: ↑ THs	Hypothyroidism ↓ Absorption of FFAs in oxidative tissues ↑ FFAs WAT absorption Thyrotoxic ↑ Absorption of oxidative tissue FFAs
Liangpunsakul et al. [18] (2003)	Human	Retrospective study	Biopsies and imaging	G1: Control G2: NASH	G2: ↓ T3	Increased risk to NASH development
Chung et al. [19] (2012)	Human	Cross sectional	Imaging	G1: Euthyroidism with NAFLD G2: Subclinical hypothyroidism with NAFLD	G2: ↓ T3	↑ NAFLD prevalence
Bano et al. [15] (2016)	Human	Prospective cohort	Imaging	G1: NAFLD Euthyroidism G2: NAFLD Hypothyroidism G3: NAFLD Hyperthyroidism	G2: ↓ THs	↑ Fibrosis ↑ Risk for NAFLD progression
Kim et al. [20] (2018)	Human	Cross sectional	Biopsies	G1: NAFLD strict-normal thyroid function G2: NAFLD low thyroid function	G2: ↓ THs	↑ Fibrosis ↑ Risk of progression to NASH
Manka et al. [21] (2019)	Human	Retrospective study	Imaging	G1: NAFLD grade 1 G2: NAFLD grade 2 G3: NAFLD grade 3 G4: NAFLD grade 4	G1: ↓ T3 G2: ↓↓ T3 G3: ↓↓ T3 G4: ↓↓↓ T3	↑ Risk of Fibrosis
Kim et al. [22] (2020)	Human	Retrospective study	Imaging	G1: NAFLD strict-normal thyroid function G2: NAFLD low thyroid function	G2: ↓ THs	↑ Fibrosis ↑ Risk for all-cause and cardiovascular mortality
D’Ambrosio et al. [23] (2021)	Human	Retrospective Single-Center study	Biopsies	G1: NASH G2: Fibrosis G3: Steatosis	G1: ↓ T3 G2: ↓↓ T3 G3: ↓↓↓ T3	↑ Risk of NAFLD progression
Du et al. [16] (2021)	Human	Retrospective study	Imaging	G1: DM2 with NAFLD without Fibrosis G2: DM2 + NAFLD + Fibrosis	G2: ↓ T3	↑ Fibrosis
Chaves et al. [17] (2021)	Human	Cross sectional	Imaging	G1: R243Q mutation of the THR-β gene G2: Their wild-type first-degree relatives	G1: ↓ THR-β	↑ Risk for NAFLD progression
Wang et al. [24] (2021)	Human	Cross sectional	Imaging	G1: Hypothyroidism G2: Hypothyroidism + NAFLD	G2: ↓ T3	↑ Liver FFAs

G1 (Group 1); G2 (Group2); G3 (Group 3); G4 (Group 4); FFA (free fatty acid); NAFLD (non-alcoholic fatty liver disease); NASH (non-alcoholic steatohepatitis); ↑ increases; ↓ decreases; ↓↓ greater decreases; ↓↓↓ severe decreases.

### 2.3. Metabolic Action of Thyroid Hormone in Liver with MAFLD

The publications that aimed to understand the mechanisms involved in thyroid hormones implicated in the progression of MAFLD were selected and organized into two tables: Table 2 (clinical studies) and Table 3 (experimental studies). 

Table 2 presents the selected clinical studies that verified the altered mechanisms of THs metabolism and their effects on the liver and on the progression of MAFLD. Designs included a cross-sectional study [25], a cohort [26], a randomized controlled trial [27] and an extension study [28]. The selected works used two MAFLD evaluation techniques. One study used imaging tests [25], while the other three performed biopsies. Regarding the study groups, we observe that all were composed of patients with or without MAFLD. Of the selected works, two studies carried out the use of MGL-3196, an analogue of THR-β [27,28] as proposed treatment. Serum THs levels were measured by biochemical tests. Interestingly, the studies demonstrated an alteration in the REDOX state and a reduction in the expression of genes positively stimulated by T3 and linked to metabolic functions in the liver [25,26]. When the THR-β analog treatment was used, the mitochondrial capacity improved, reducing liver fat and decreasing the risk of progression to other stages of MAFLD [27,28].

The selected experimental studies are shown in Table 3; these studies explore the mechanisms of TH metabolism and its relationship with MAFLD. The selection features different experimental designs. Four studies used a cell model [29,30,31,32], while the others used an animal model, demonstrating different ways of inducing MAFLD. The cell studies used the administration of FFAs [29] or palmitic and oleic acid [30]. The animal models show heterogeneity regarding the induction of the model, but the vast majority used ae high fat diet (HFD) [33,34,35,36,37,38,39,40]; one study used a pork-derived diet [41], while other models did not induce MAFLD but induced situations of hypo and hyperthyroidism [42,43,44,45,46,47,48,49], subsequently evaluating its effects on the liver. Most of the selected articles included some type of hormone supplementation. Some studies used T2, T3 and T4 hormones [29,30,31,32,34,35,38,39,42,45,46,50,51], while others used THR analogs [35,38,39,52]. One study used soy oil and fish oil supplements [48] and one study used an antioxidant compound [40], whereas other studies worked with gene mutations [52,53,54].

We observe that the data described in these studies relates to three main mechanisms. The first is the inflammatory process, which is due to a high-fat diet and low levels of physical exercise. An excess of fat in hepatocytes generates a signaling cascade of pro-inflammatory factors such as IL-6 and TNF-α. At the same time, an imbalance between antioxidant defenses and reactive oxygen species generates an altered (pro-oxidative) REDOX state, characterizing the second process. These disorders negatively affect the activity of enzymes capable of activating THs, decreasing the activity of type 1 deiodinase (Dio1), responsible for converting T4 to T3, and increasing the activity of type 3 deiodinase (Dio3), responsible for inactivating T3. This results in all mechanisms dependent on T3 levels in the liver being suppressed. This reduced signaling process decreases the activation of genes responsible for the metabolism of lipids and carbohydrates in the liver and causes systemic changes such as increased serum levels of cholesterol and triglycerides, and hepatic changes such as reduced lipid catabolism, reduced β- oxidation and increased lipid synthesis. The set of metabolic imbalances in the liver, secondary to these altered mechanisms, significantly increases the risk of developing MAFLD, leading to fibrosis, cirrhosis and, in more severe cases, hepatocarcinoma.

**Table 2:** Metabolic action modulated by thyroid hormone signaling in clinical studies.

Manuscript	Sample	Study Design	NAFLD Assessment Technique	Groups	Treatment	Dose	THs	TH Target	Effect on Liver
Mustafa et al. [25] (2009)	Human	Cross sectional	Imaging	G1: Control G2: NASH	—	—	G2: ↓ T3	↑ serum MDA ↑ serum NO ↓ serum GSH ↓ serum GPx	↑ Risk NASH progression
Krause et al. [26] (2018)	Human	Cohort Study	Biopsies	G1: NAFLD	—	—	G1: ↓ T3	↓ Dio1 mRNA ↓ THR-β mRNA	↑ Hyperlipidemia ↑ Risk NASH progression
Harrison et al. [27] (2019)	Human	Randomized Controlled Trial	Biopsies	G1: NASH + Placebo G2: NASH + MGL-3196	MGL-3196	80 mg	G2: ↑ THR-β	THR-β	↓ Hepatic fat Restoration of Mitochondrial function
Harrison et al. [28] (2021)	Human	Extension Study	Biopsies	G1: NASH	MGL-3196	80 or 100 mg	G1: ↑ T3 and ↓ rT3	THR-β	↓ Risk NASH progression

G1 (Group 1); G2 (Group2); G3 (Group 3); G4 (Group 4); G5 (Group 5); FFA (free fatty acid); NAFLD (non-alcoholic fatty liver disease); NASH (non-alcoholic steatohepatitis); Dio1 (iodothyronine deiodinase 1); GSH (reduced glutathione); THRβ (thyroid hormone receptor-beta); MDA (malondialdehyde); GPx (glutathione peroxidase); NO (nitric oxide); ↑ increases; ↓ decreases.

**Table 3:** Metabolic actions modulated by thyroid hormone signaling in experimental studies.

Manuscript	Sample	Groups	Treatment	Dose	THs	TH Target	Effect on Liver
Nozaki et al. [31] (1992)	Cells	HepG2 cells	T3	0.1/μg/mL 0.2/μg/mL 0.3/μg/mL	↑ T3	↑ HTGL mRNA ↑ Hepatic lipid hydrolysis	↑ Lipogenesis
Zhang et al. [32] (2004)	Cells	HepG2 cells with Luciferase Vectors-CPT-1	T3	100 nM	↑ T3	↑ PGC-1α mRNA ↑ CPT-1α mRNA	↑ Fatty acid β-oxidation
Grasselli et al. [29] (2011)	Cells	G1: Control G2: FFAs G3: FFAs + T2 G4: FFAs + T3	T2 T3	10^−7^ to 10^−5^ M 10^−7^ to 10^−5^ M	G3: ↑ T2 G4: ↑ T3	↓ PPAR-δ and -γ mRNA ↓ SOD ↓ CAT	↓ Excess fat ↓ TAG
Grasselli et al. [30] (2014)	Cells	G1: Control cells G2: Hepatoma cell + oleate/palmitate	T2 T3	10^−6^ M to 10^−5^ M	G2: ↑ T3	↑ UCP2 mRNA ↑ CPT-1 mRNA ↑ UCP2 protein ↑ CPT1 protein ↑ ROS ↓ CAT ↓ GSH	↓ Extracellular TAG ↑ Fatty-acid oxidation ↓ NAFLD progression
Ness et al. [46] (1990)	Rats	G1: Normal G2: Hypophysectomized	T3	10 μg/100 g	G2: ↑ T3	↑ CYP7A1 mRNA	↑ Cholesterol metabolism
Huang et al. [44] (1998)	Rats	G1: Hypothyroidism G2: Hyperthyroidism G3: Euthyroidism	—	—	G1: ↓ THs G2: ↑ THs	Hyperthyroid ↑ Acetyl-CoA carboxylase mRNA Hypothyroid ↓ Acetyl-CoA carboxylase mRNA	Hyperthyroid ↑ Hepatic lipogenesis Hypothyroidism ↓ Hepatic lipogenesis
Feng et al. [43] (2000)	Mice	G1: Control G2: Hyperthyroid	—	—	G2: ↑ T3 + THR	↑ G6PC ↑ PCK1	↑ Glycogenolysis ↑ Gluconeogenesis
Jackson-Hayes et al. [53] (2003)	Mice	Transgenics (CPT-1α-luciferase) with/without the 1st intron of the CPT-1α gene	—	—	↑ T3 + THR	↑ CPT-1α gene	↑ Fatty acid β-oxidation
Noguchi-Yachide et al. [49] (2007)	Mice	G1: Euthyroidism G2: Thyrotoxic G3: Hypothyroidism	—	—	G2: ↑ T3 + THR	↑ LXR-α mRNA ↑ CYP7A1 mRNA	Lipid homeostasis
Liu et al. [52] (2007)	Mice	G1: WT control G2: Mutation in THR	—	—	G2: ↓ T3 + THR	↓ PPARα protein	↓ Fatty acid β-oxidation
Lopez et al. [45] (2007)	Rats	G1: Normal G2: Hypophysectomized G3: Thyroidectomy	T3	10 μg/100 g	G3: ↑ T3	↑ LDL receptor mRNA	↑ FFA absorption
Cable et al. [55] (2009)	Rats	G1: NASH + Vehicle G2: NASH + T3 G3: NASH + MB07811	MB07811 T3	1–50 mg/kg/day 650 μg/kg/day	G2: ↑ T3 G3: ↑ T3 + THR	↑ CPT-1 mRNA ↑ PGC-1α mRNA	↑ Mitochondrial β-oxidation
Mollica et al. [38] (2009)	Rats	G1: Control G2: HFD G3: HFD + T2	T2	25 μg/100 g	G3: ↑ T2	↑ PPAR-α ↑ CPT-1	↑ mitochondrial respiration ↓ degree of steatosis
Adams et al. [50] (2010)	Mice	G1: C57BL/6 control G2: C57BL/6 with T3	PBS T3	500 μg/kg	G2: ↑ T3	↑ FGF21 mRNA	↑ lipolysis ↑ hepatic fatty acid oxidation
Sousa et al. [48] (2011)	Rats	G1: Euthyroidism G2: Hypothyroidism	Soybean oil Fish oil	0.5 mL	G2: ↓ T3	↑ PPARα protein ↓ D1 mRNA	↓ Serum triglycerides ↓ Hepatic TAG levels
Grasselli et al. [34] (2012)	Rats	G1: DP G2: HFD G3: HFD + T2 G4: DP + T2	T2	25 μg/100 g	G3: ↑ T2 G4: ↑ T2	↑ PPARγ mRNA ↑ acyl-CoA oxidase mRNA	↓ Inflammation ↓ Adipose triglyceride lipase ↑ FFA oxidation
Santana-Farré et al. [47] (2012)	Rats	G1: Neonatal Hypothyroidism G2: Age-matched euthyroid G3: Euthyroid weight paired	—	—	G1: ↓ T3	↑ PPARα mRNA ↓ LXR mRNA ↓ CD36 mRNA ↓ genes uptake Ags	↓ Absorption of FFAs in the liver
Cavallo et al. [42] (2013)	Rats	G1: Euthyroid G2: Hypothyroid G3: Hypothyroid + T2	T2	150 µg/100 g	G3: ↑ T2	↑ CPT-1 protein ↑ OXPHOS	↑ Fatty acid β-oxidation ↓ Adiposity ↓ Dyslipidemia
Alonso-Merino et al. [51] (2016)	Cells Rats	G1: Euthyroid G2: Hyperthyroid	T4 T3	T4 7 ng/g T3 35 ng/g	G2: ↑ T3 + THR	↓ TGF-β mRNA	↓ Fibrosis progression
Iannucci et al. [35] (2017)	Rats	G1: Control G2: HFD G3: HFD + T2 G4: HFD + T3	T2 T3	25 µg/100 g 2.5 µg/100 g	G3: ↑ T3 + THR G4: ↑ T3 + THR	↑ CPT-1α protein ↑ UCP2 protein ↑ p-ERK protein ↑ p-Akt protein	↑ Lipolysis ↑ Autophagy ↑ Fatty acid β-oxidation
Senese et al. [39] (2017)	Rats	G1: Control G2: HFD G3: HFD + T2 G4: HFD + T3	T2 T3	25 μg/100 g^−1^ 2.5 μg/100 g^−1^	G3: ↑ T2 G4: ↑ T3	↑ Dio1 mRNA ↑ THRβ mRNA	↓ TAG ↓ Lipogenesis ↑ Fatty acid oxidation
Bruinstroop et al. [56] (2018)	Rats	G1: Control G2: MCD diet	—	—	G2: ↓ T3	↓ T3 hepatic ↓ Dio1 mRNA	↑ NAFLD progression
Xia et al. [40] (2019)	Mice	G1: C57BL/6 control G2: C57BL/6 HFD G3: C57BL/6 HFD + Myr	Myricetin	100 mg/kg^−1^	G3: ↑ T4 and ↑ T3	↑ Dio1 mRNA ↑ Dio1 protein ↑ Dio1 activity ↑ THRβ mRNA ↑ THRβ protein	↓ Hepatic steatosis ↑ Lipid metabolism
Luong et al. [37] (2020)	Rats	G1: Control G2: HFD G3: HFD + MGL-3169 (5.0 mg/kg) G4: HFD + MGL-3169 (1.5 mg/kg) G5: HFD + MGL-3169 (0.5 mg/kg) G6: HFD + T3 (0.5 mg/kg)	MGL-3196 T3	0.5–5.0 mg/kg	G3: ↑ T3 and ↑ THR G4: ↑ T3 and ↑ THR G5: ↑ T3 and ↑ THR G6: ↑ T3 and ↑ THR	↑ Dio1 mRNA ↑ Me1 mRNA	↓ Serum lipid profile ↑ FFA oxidation
Bruinstroop et al. [54] (2021)	Mice	G1: Control NCD G2: Control WDF G3: Dio1 LKD WDF	—	—	G3: ↓ T3	↓ Dio1 mRNA ↓ Dio1 activity	↑ TAG ↑ Cholesterol ↑ Risk for NAFLD progression
Caddeo et al. [33] (2021)	Mice	G1: C57BL/6 G2: C57BL/6 + HFD G3: C57BL/6 + HFD + MGL-3196 G4: C57BL/6 + HFD + TG68	MGL-3196 TG68	3 mg⋅kg^−^^1^ 2.8 mg⋅kg^−^^1^	G3: ↑ T3 G4: ↑ T3	↑ Dio1 mRNA ↑ THRsp mRNA	↓ liver weight ↓ Serum TAG ↓ Plasma ALT ↓ Plasma AST
Kannt et al. [36] (2021)	Mice	G1: C57BL/6J + DP G2: C57BL/6J + HFD G3: C57BL/6J + HFD + Resmetirom	Resmetirom	3 mg·kg^−1^	G3: ↑ THR	↑ Dio1 mRNA ↑ CYP7A1 mRNA ↑ Me1 mRNA	↓ Serum lipid profile ↓ Liver weight ↓ NAFLD Score
Ge et al. [41] (2022)	Mice	G1: C57BL/6 control G2: C57BL/6 LOP G3: C57BL/6 HOP G4: C57BL/6 LOP + Dityr G5: C57BL/6 Dityr	—	—	G2: ↓ T3 G3: ↓ T3 G4: ↓ T3 G5: ↓ T3	↓ Dio1 mRNA ↓ THRβ mRNA ↓ CPT-1α mRNA ↓ PPARα mRNA ↓ PGC-1α mRNA ↓ CYP7A1 mRNA ↑ MDA ↑ ROS ↓ CAT ↓ GSH/GSSG	↑ Risk NAFLD ↑ Inflammation ↑ Oxidative stress ↓ Hepatic energy metabolism ↑ Hepatic lipid synthesis ↓ Hepatic lipid catabolism ↓ Fatty-acid oxidation

G1 (Group 1); G2 (Group2); G3 (Group 3); G4 (Group 4); G5 (Group 5); FFA (free fatty acid); NAFLD (non-alcoholic fatty liver disease); NASH (non-alcoholic steatohepatitis); HTLG (hepatic triacyiglycerol lipase); PGC-1α (peroxisome proliferator-activated receptor-gamma coactivator-1alpha); CPT-1α (carnitine palmitoyltransferase-1alpha); SOD (superoxide dismutase); CAT (catalase); CYP7A1 (cholesterol 7-alpha-monooxygenase); G6PC (glucose-6-phosphatase); PCK1 (phosphoenolpyruvate carboxykinase 1); LXR-α (liver X receptor-alpha); PPAR-α (peroxisome proliferator-activated receptor-alpha); FGF21 (fibroblast growth factor 21); Dio1 (iodothyronine deiodinase 1); UCP2 (uncoupling protein 2); ROS (reactive oxygen species); GSH (reduced glutathione); GSSG (oxidized glutathione); TGF- β (transforming growth factor-beta); ERK (extracellular signal-regulated kinases); Akt (protein kinase B); THRβ (thyroid hormone receptor-beta); Me1 (malic enzyme 1); MDA (malondialdehyde); GPx (glutathione peroxidase); NO (nitric oxide); ↑ increases; ↓ decreases.

## 3. Discussion

### 3.1. THs Dependent Mechanisms in Hepatic Metabolism 

**Figure 2:**
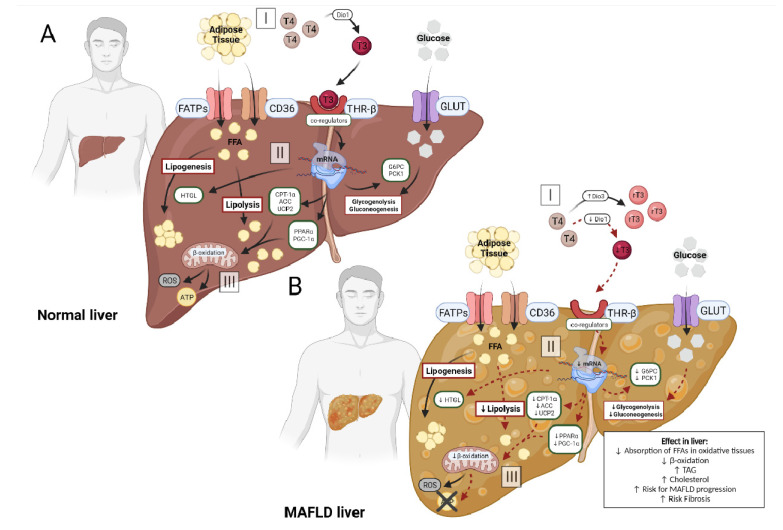
(**A**): In the normal liver, circulating adipose tissue enters the hepatocyte via specific receptors, FATPs and CD36 (A-I). At the same time, the circulating hormone T4 is converted into T3 by the enzyme Dio1, making it biologically active and enabling its binding to its THRβ receptor (A-I). This attachment generates the activation of co-regulators, which could stimulate the transcription of genes in the hepatocyte (A-II). The main genes activated in this process are CPT-1α, UCP2, PGC-1α and PPARs, increasing hepatic lipolysis and β-oxidation, and ATP production (A-III). Other genes that are indirectly stimulated are HTLG linked to the process of lipogenesis and genes linked to glycogenolysis and gluconeogenesis, G6PC and PCK-1, using the serum glucose that entered the hepatocyte via the specific transporter GLUT (A-II). (**B**): In the MAFLD liver, the process is changed. Circulating adipose tissue enters the hepatocyte via FATPs and CD36 (B-I). However, circulating T4 does not convert into T3, but into rT3 or T2, due to the reduction of Dio1 activity and the increase of Dio3, decreasing the binding of the hormone to the THRβ (B-I) receptor. With lower availability of T3, the activation of co-regulators does not occur effectively, reducing the transcription of genes in the hepatocyte (B-II). Among the affected genes are CPT-1α, UCP2, PGC-1α and PPARs, decreasing hepatic lipolysis and β-oxidation, reducing ATP production and increasing reactive oxygen species (ROS) (B-III). Other genes also affected alter the process of lipogenesis and decrease glycogenolysis and gluconeogenesis (B-II). This imbalance of hepatic metabolism is one of the main factors involved in the progression of MAFLD, with a high risk of fibrosis.

The processes involving hepatic metabolism occur through the action of several genes, and the modulation of these genes is performed by the signaling of THs via THR-β isoform in the liver, generating signaling of coactivators and corepressors, co-regulators of gene transcription [3,57,58,59] (Figure 2A). 

The signaling generated by THs is one of the central mechanism of changes in liver functions. The mobilization of proteins responsible for the absorption of free fatty acids (FFAs) is an important source of lipids in the liver. Its absorption is carried out via specific transport proteins, such as fatty acid transport proteins (FATPs) and translocase protein (CD36) [60]. Studies have suggested that normal amounts of circulating THs facilitate the absorption of FFAs by tissues, thus establishing a direct association between FFA transporters and THs [16,61,62].

In addition, THs stimulate gene transcription of mitochondrial β-oxidation-linked proteins, such as carnitine palmitoyltransferase-1 (CPT-1), coactivator 1 α (PGC1-α) and peroxisome proliferator-activated receptor gamma (PPARs) [63,64,65]. Changes in T3 availability directly impact the ability to metabolize FFAs, reducing the mRNA of these proteins and increasing the risk of developing metabolic dysfunction associated with fatty liver disease (MAFLD). It is suggested that T3 plays a central role in the regulation of mitochondrial β-oxidation, which is an important step in energy (Table 2 and Table 3). The hepatic glycolytic pathway is also regulated by THs, involving gluconeogenesis and glycogenolysis [61]. In this context, changes in hepatic glucose metabolism are observed with altered circulating levels of THs [62]. In situations of hyperthyroidism, there is a resistance to insulin status, probably due to increased expression of CPT-1 and reduced malonyl-CoA. It is known that malonyl-CoA in the liver, has the ability to inhibit CPT-1 by increasing circulating glucose-stimulated insulin release [66].

Another factor that may explain the increase in circulating glucose levels is the action of serum T3 in the activation of genes involved in gluconeogenesis. Increased activity of enzymes such as glucose-6-phosphatase (G6PC), along with phosphoenolpyruvate carboxykinase 1 (PCK1) and pyruvate carboxylase (PC) are associated with increased gluconeogenesis activity. Studies have shown that higher levels of serum T3 increase the transcription of the G6PC and PCK1 genes, involved in the homeostatic control of glucose levels via gluconeogenesis (Table 2 and Table 3).

Some limitations were observed in this systematic review. First, clinical studies aimed at verifying the risk of developing MAFLD in patients with thyroid dysfunction that is already established. Well-designed studies on euthyroid patients can bring new answers about the role of THs on euthyroid and the possible causes of the onset of MAFLD. Another important point seen here is the lack of studies exploring the complexity of the involvement of THs on the progression of MAFLD, especially clinical studies that performed a biopsy for the diagnosis of the disease.

The data compiled in this systematic review may suggest that the availability of THs directly impacts hepatic metabolic capacity, given the importance of THs signaling in the regulation of metabolic pathways. The key role played by THs seems to be fundamental to the understanding of the triggering and progression of liver metabolic diseases and could be involved as part in the treatment of this disease.

### 3.2. Thyroid Hormone Metabolism Alterations and MAFLD

It is known that in normal situations hepatocytes show high expression of Dio1 and low expression of Dio3, maintaining adequate TH function and hepatic metabolism activity (Figure 2A). Recently, evidence has shown that alterations in these enzymes results in changes in the availability of hepatic T3 (Figure 2B). The study by He et al. (2017) found, in a meta-analysis, evidence of a direct and significant association of low T3 levels with a higher risk of developing MAFLD, when compared with normal thyroid function [13]. 

Table 2 presents the clinical studies selected for review. The design used in the studies is mainly cross-sectional or cohort. Studies with this design answer specific questions, generating limited data related to the mechanisms involved in thyroid metabolism in MAFLD. Randomized clinical trials [27,28] are more robust. However, studies that used the biopsy technique for the diagnosis of MAFLD somehow drew attention. The tissue fragment collected could have been better explored, answering questions at the molecular level that have not yet been answered in clinical studies. We believe that new studies using this approach should evaluate gene expression and quantification of proteins related to the mechanisms involved in the disease, thus creating new data related to this topic. 

In general, all studies reached the same conclusion. All, including our work, confirmed a direct association between serum T3 levels and MAFLD progression. However, our study advances knowledge, bringing different insight from the selected studies. Our work was able to better understand and correlate the mechanisms that are influenced by a lower availability of T3 in the liver. 

Our findings show that alterations in the expression of genes involved in T3 activation have a negative impact on hepatic metabolic capacity. A reduction of Dio1 expression [26] reduces the availability of T3, affecting the binding of the hormone with its THR-β receptor, which decreases the activation of other genes involved in hepatic metabolism. These changes generate a reduction in mitochondrial capacity and, consequently, a dysfunction in hepatic lipid metabolism, increasing fat deposits and increasing the risk of MAFLD progression. In contrast, other studies found that an increase in the amount of the THR-β receptor increases the sensitivity of the liver tissue to the T3 hormone, improving mitochondrial function, β-oxidation, and slowing the progression of the disease.

The findings of the studies listed in Table 3 present similar results. They reiterate the association between liver T3 availability and MAFLD development and progression. THs regulate genes linked to the functionality of several metabolic pathways, such as lipogenesis, lipid oxidation and hepatic gluconeogenesis [6]. Table 3, together with Figure 2B, clearly demonstrates that the reduction of T3-stimulated genes favors MAFLD progression. Reduced expression of PPAR-α reduces the transcription of genes involved in lipid homeostasis and, together with lower expression of CPT-1, generates a reduction in the translocation of FFA from the cytosol to the mitochondrial matrix, decreasing the metabolism of fat and the mitochondrial capacity [67]. In addition, other genes that are inhibited by T3 inactivity stimulate disease progression, as the Me1 gene, that decreases the encoding of cytosolic enzymes linked to fatty acid biosynthesis and the reduction of UCP2, decreases the decoupling of oxygen consumption from ATP synthesis.

The findings of this review support the hypothesis that as the disease progresses to more severe conditions such as NASH and fibrosis, Dio1 expression decreases while Dio3 expression increases, decreasing the availability of active T3 (Figure 2B-I). Local T3 reduction has a negative impact on the activation of co-regulators released by THRs, causing a cascade effect on genes involved in lipolytic processes: lower transcription of PPARs, CPT-1 and PGC1-α (genes involved in β-oxidation processes) (Figure 2B-II). In addition to the lipolytic pathway, the glycolytic pathway undergoes changes, with reduced transcription of G6PC and PCK1 (Figure 2B-II). It leads to an increase in the accumulation of triglycerides by hepatocytes and the reuptake of LDL, due to an impairment of the breakdown of triglycerides and a lower capacity for β-oxidation of FFAs, reducing energy production (Figure 2B-III).

Nevertheless, there are still several gaps in knowledge about MAFLD. For example, knowing that the pathophysiology of MAFLD is characterized by inflammatory changes and the REDOX state, it is possible to think that the use of antioxidants could improve the dysfunction in thyroid hormone metabolism, improving mitochondrial capacity and consequently lipid metabolism, probably stabilizing MAFLD.

## 4. Methods

### 4.1. Protocol and Registration

This systematic review adheres to the PRISMA guidelines and was registered in the International Prospective Register of Systematic Reviews (PROSPERO CRD42022323766).

### 4.2. Study Objectives

Our objective was to investigate the role of thyroid hormone metabolism in MAFLD. We focused on the following research question:

What are the mechanisms of THs that are associated with MAFLD progression?

### 4.3. Eligibility Criteria

We included only cohort studies (prospective or retrospective), clinical trials and experimental studies that related THs levels and/or mechanisms with MAFLD. Exclusion criteria were age younger than 18 years and articles that were not a cohort study, clinical trial or experimental. Only articles written in English were considered. No restrictions on publication date were applied.

### 4.4. Search Strategy and Study Selection

We performed a systematic search of the MEDLINE (via PubMed) and Embase databases from their inception to March 2022. Comprehensive search queries included text words and descriptors (MeSH and Entree) based on the phrases ‘non-alcoholic fatty liver disease’ and ‘thyroid hormones’. 

The complete search strategy for Embase and Pubmed was: Pubmed Exposition: (“Thyroid hormones” [Mesh] OR “Thyroid Hormone Receptors beta” [Mesh]) OR (“iodothyronine deiodinase type I” [Supplementary Concept] OR “iodothyronine de-iodinase type II” [Supplementary Concept] OR “iodothyronine deiodinase type III” [Supplementary Concept]) AND (“Non-alcoholic Fatty liver disease” [Mesh] OR “Fatty liver” [Mesh] OR “Diet, High-Fat” [Mesh]) AND (“Clinical Trial” [Publication Type] OR “Clinical Study” [Publication Type] OR “Observation” [Mesh] OR “Randomized controlled trial” [Publication Type] OR “Rats” [Mesh] OR “Mice” [Mesh] OR “Cells” [Mesh]) NOT (“Review” [Publication Type] OR “Systematic review” [Publication Type] OR “Meta-Analysis” [Publication Type]). Embase: Exposition: ‘Thyroid hormones’ OR ‘Thyroid Hormone Receptors beta’ OR ‘deiodinase type 1’ OR ‘de-iodinase type 2’ OR ‘deiodinase type 3’ AND ‘Nonalcoholic Fatty liver/exp’ OR ‘Non-alcoholic Fatty liver’ OR ‘Fatty liver’ OR lipid diet’ AND ‘Clinical Trial’ OR ‘Clinical Study’ OR ‘Observation study’ OR ‘Randomized controlled trial’ OR ‘Rat’ OR ‘Mouse’ OR ‘Cells’.

Three independent reviewers (RAM, FA and RTR) assessed records for inclusion based on titles and abstracts. Abstracts that did not meet the inclusion criteria or that met the exclusion criteria were discarded. The remaining records and abstracts that did not provide enough information to decide on their exclusion were selected for full-text evaluation, which was performed by the same reviewers independently. A fourth reviewer (SW) resolved the disagreements.

### 4.5. Data Collection and Extraction

Independent reviewers extracted the data using a standardized system. The following information was obtained: first author, year of publication, sample, study design, NAFLD assessment technique, groups, treatment, dose, THs, TH target, effect in liver. The research team verified and discussed the results of the extraction.

## 5. Conclusions 

There have been advances in the understanding of the mechanisms involving THs and THRs in the maintenance of liver homeostasis in recent years. The interrelationship between the availability of tissue T3 and the signaling of THRs in the metabolic processes involved in liver diseases seems to be the key to better understand the disease. Recent findings have given us a basis of knowledge on lipid metabolism and its complexity in the processes of FFA regulation, although its relationship with THs in the disease is still unclear. Deepening knowledge of the mechanisms involved in MAFLD related to THs seems to be the focus for future studies.
